# Temporal and Quantitative Analysis of Aortic Immunopathologies in Elastase-Induced Mouse Abdominal Aortic Aneurysms

**DOI:** 10.1155/2021/6297332

**Published:** 2021-11-16

**Authors:** Kangli Tian, Congcong Xia, Haole Liu, Boyu Xu, Panpan Wei, Weilai Fu, Ming Lu, Yankui Li, Yafeng Li, Daxin Cheng, Enqi Liu, Sihai Zhao

**Affiliations:** ^1^Institute of Cardiovascular Science, Basic Medical School of Xi'an Jiaotong University, Xi'an, Shaanxi 710061, China; ^2^Laboratory Animal Center, Xi'an Jiaotong University School of Medicine, Xi'an, Shaanxi 710061, China; ^3^Department of Vascular Surgery, The Second Hospital of Tianjin Medical University, Tianjin 300211, China

## Abstract

**Objective:**

Elastase-induced abdominal aortic aneurysm (AAA) model is widely used for aneurysmal pathogenesis and translational research. However, temporal alternations in aneurysmal histologies remain unknown. This study is aimed at analyzing temporal immunopathologies of aneurysmal aorta following experimental AAA induction.

**Methods:**

Male C57BL/6J mice at the age of 10-14 weeks received intra-aortic infusion of elastase to induce AAAs. Aortic diameters at the baseline and indicated days after AAA induction were measured, and aortae were collected for histopathological analysis.

**Results:**

Aorta diameters increased from 0.52 mm at the baseline levels to 0.99 mm, 1.34 mm, and 1.41 mm at days 7, 14, and 28, respectively, corresponding 90%, 158%, and 171% increases over the baseline level. Average aortic diameters did not differ between days 14 and 28. Severe elastin degradation and smooth muscle cell depletion were found at days 14 and 28 as compared to the baseline and day 7. No difference in the scores of medial elastin and SMC destruction was noted between days 14 and 28. Consistent results were found for leukocyte accumulation, neoangiogenesis, and matrix metalloproteinase expression. Twenty-eight days after AAA induction, all aneurysmal pathologies showed an attenuated trend, although most histopathological parameters did no differ between days 14 and 28.

**Conclusion:**

Our data suggest that almost aneurysmal immunohistopathologies reach maximal 14 days following AAA induction. Analysis of day 14 histologies is sufficient for AAA pathogenesis and translational studies in elastase-induced mouse experimental AAAs.

## 1. Introduction

Abdominal aortic aneurysms (AAAs) are the localized dilatations of abdominal aortic segments defined as a more than 50% increase over the proximal segment or aortic diameter over 3.0 cm [1–3]. Asymptomatic aneurysm rupture is lethal with the mortality of up to 90%. Effective clinical AAA management is required to limit further progression of small AAAs. However, the precise mechanisms by which AAAs enlarge remain largely unclear and thus impeding therapeutical strategies.

Animal models are important for understanding AAA pathogenesis and evaluating therapeutical safety and efficacy. Currently, used AAA models include infrarenal aortic infusion of porcine pancreatic elastase (PPE), periaortic calcium chloride painting, subcutaneous infusion of angiotensin II (Ang II), and genetically or pharmacologically modified animals for transforming growth factor-*β* and its receptors [4–7]. Main AAA pathological features include elastin degradation, smooth muscle cell (SMC) depletion, leukocyte accumulation, and angiogenesis. In the PPE model, PPE infusion induces aortic leukocyte infiltration, degrades medial elastin, and leads to SMC loss due to apoptosis, thus disrupts aortic architecture and promotes AAA formation and progression [5, 8–11]. Because of highly histological similarity between PPE-induced and clinical AAAs, the PPE model has been increasingly used in AAA research [8, 12–15]. In published studies, histological examinations have been limited to one time point analysis such as day 14 or day 28 following AAA induction [9, 16–18]. It is not known whether one time point analysis at day 14 or 28 represents characteristics of AAA pathologies. Thus, it warrants temporal analysis of aneurysmal histopathologies for better understanding of AAA pathogenesis and translational studies.

In this study, we collected aortae prior to day 0 and indicated days after AAA induction and performed a temporal and quantitative immunopathological analysis of experimental AAAs induced by elastase infusion.

## 2. Materials and Methods

### 2.1. Mice

Male C57BL/6J (10 to 14 weeks old) mice were obtained from the Laboratory Animal Center of Xi'an Jiaotong University, Xi'an, China. All mice were housed and maintained in specific pathogen free animal facility of Xi'an Jiaotong University with a 12 h/12 h light-dark cycle. Animal experimental protocols were approved by the Laboratory Animal Administration Committee of Xi'an Jiaotong University, and all studies were performed according to the Guidelines for Animal Experimentation of Xi'an Jiaotong University (protocol No. 2019-1178).

### 2.2. Creation of Experimental AAAs

AAAs were created in mice via transient intraluminal infusion of PPE as previously reported [8–11, 19]. Type I PPE for infusion was freshly prepared in phosphate-buffered saline (PBS) (1.5 U/mL, Cat # E-1250; Sigma-Aldrich, St. Louis, USA). Briefly, under anesthesia with isoflurane inhalation, abdominal cavity was opened, and infrarenal aorta was exposed and isolated from the level of the left renal vein to the iliac bifurcation. Within infusion region, all visible arterial branches were ligated. After temporarily ligated infrarenal aorta, aortotomy was created using a 30-gauge needle, and heat-tapered P-10 tubing was inserted into the controlled segment. Under constant pressure, 30 *μ*L of PPE solution was infused for 5 minutes. After PPE infusion, the PE-10 tubing was withdrawn, the aortotomy was closed with 11-0 suture (Lingqiao, Ningbo, China), and aortic flow was restored. Following 2 hours recovery, mice were housed in separate cages with free access to food and water.

### 2.3. Aortic Diameter Measurements

External maximal aortic diameters were measured prior to PPE infusion or 7, 14, or 28 days following PPE infusion. Briefly, exposed infrarenal aorta was photographed under a surgical microscope equipped with a digital camera (ProS5 Lite, Motic, China) and measured with the Images Plus 3.0 ML software (Motic, China). An AAA was defined as a 50% or greater increase in aortic diameter over the baseline level [9].

### 2.4. Histochemistry

Abdominal aortae were harvested and immediately embedded in optimal cutting temperature compound for following analysis. Aorta was cut into 6 *μ*m thick serial sections for hematoxylin and eosin (H&E) and elastic van Gieson (EVG) staining. Elastin degradation was graded I to IV as previously reportedly [8, 11, 20].

### 2.5. Immunohistochemistry (IHC)

A biotin-streptavidin-peroxidase procedure was used to stain SMCs, identify leukocyte subsets, and assess aortic angiogenesis. Primary antibodies used in this study included anti-SMC alpha-actin (1: 200; Cat # NB300-978; Novus Biologicals, Centennial CO), anti-CD68 (macrophages, 1: 200; CD68, Cat # 137002; BioLegend, San Diego, CA), anti-CD4 mAb (CD4^+^ T cells, 1 : 200; Cat # 100402; BioLegend), anti-CD8 mAb (CD8^+^ T cells, 1 : 200; Cat # 100702; BioLegend), anti-B220 mAb (B cells, 1 : 100; CD45R, Cat # 103202; BioLegend), anti-CD31 mAb (mural angiogenesis, 1 : 200; Cat # 100402; BioLegend), anti-matrix metalloproteinase (MMP) 2 antibody (1 : 200; Cat # AF1488, R&D Systems, Minneapolis, MN), and MMP9 (1 : 200; Cat # AF909, R&D Systems). Secondary antibodies and other reagents were biotinylated goat anti-rat antibody (1 : 400; Cat # BA-9400; VECTOR, Burlingame, CA), donkey anti-goat IgG antibody (1 : 400; Cat # 705-065-003; Jackson ImmunoResearch Inc., West Grove, PA), streptavidin-peroxidase conjugate (Jackson Immuno Research Laboratories (1 : 400; Cat # 016-030-084)), and AEC substrate kit (Cat # SK-4200, VECTOR).

Medial SMC loss and aortic macrophage accumulation were graded as I (mild) to IV (severe) as previously reported [8, 11, 20]. Aortic accumulation of CD4^+^ T cells, CD8^+^ T cells and B200^+^ B cells were quantitated as the number of subset-mAb positively stained cells per aortic cross section (ACS). Aneurysmal angiogenesis was reported as numbers of CD31-positive vessels per ACS. For quantification of MMP 2 and 9 expression levels, the stained sections were photographed under a microscope equipped with a digital camera and positive stained area was measured with the image analysis software (WinRoof 6.5, Mitani Co. Ltd., Tokyo, Japan).

### 2.6. Statistical Analysis

Data are expressed as mean ± standard error (SE) or media ± 95%confidence interval (CI) for normally and not normally distributed variables, respectively. The Shapiro-Wilk normality test was used to determine whether a data set was normally distributed. For normally distributed data, ordinary one-way ANOVA analysis was conducted, followed by multiple comparisons were used to determine the significance between different groups. For data failing normal distribution, differences were tested using the nonparametric Kruskal-Wallis test. All statistical analyses were performed by PRISM 7.0, and *P* < 0.05 was considered significant.

## 3. Results

### 3.1. Aortic Enlargement

**Figure 1:**
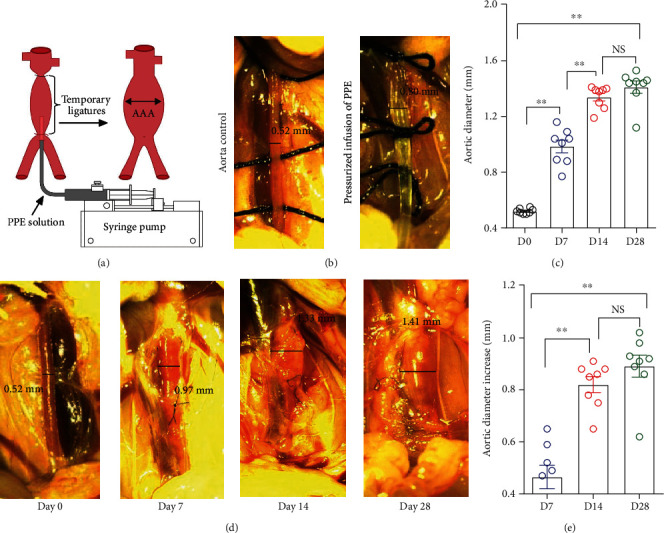
Aortic diameters in mice before and after porcine pancreatic elastase (PPE) infusion. (a) Schematic diagram of the PPE infusion procedure (modified from Pyo et al. J Clin Invest, 2000, 105 : 1641-9 [5]). (b) Aorta prior to and immediately after PPE infusion. (c) Mean and standard error of external aortic diameters at the baseline level (day 0) and indicated days after PPE infusion. (d) Representative aortic images at the baseline level (day 0) and indicated days after PPE infusion. (e) Delta changes in external aortic diameters at different time points after PPE infusion. One-way ANOVA followed by two-group comparison, ^∗∗^*P* < 0.01 between two groups. *n* = 8 mice in each group. NS: not significant difference; D: day.

As shown in Figure 1, the surgery for PPE infusion was performed in mice as previously described [8–11]. Among 32 mice, 24 mice survived after the surgery. In our mouse cohort of 10-14-week-old male C57BL/6J, average baseline aortic diameter in mice was 0.52 mm. PPE infusion increased aortic diameters to 0.99 mm, 1.34 mm, and 1.41 mm at days 7, 14, and 28, respectively (Figure 1(c)). On day 7, diameters were significantly larger as compared to the baseline level. Delta changes in aortic diameters over the baseline were 0.82 and 0.89 mm on days 14 and 28, respectively (Figures 1(b)–1(e)). All mice developed AAAs within 14 days following PPE infusion. No further significant increase in aortic diameter was observed on day 28 as compared to those on day 14 (Figures 1(c)–1(e)). These results suggest that aortic expansion induced by PPE infusion reaches the plateau on day 14 after AAA induction.

### 3.2. Destruction of Medial Elastin and SMCs

**Figure 2:**
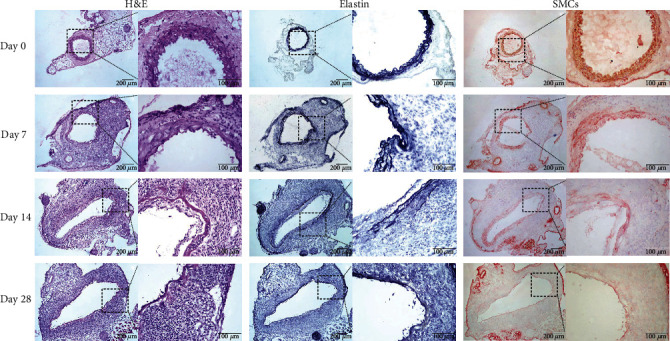
Representative images for H&E, medial elastin, and smooth muscle cells (SMCs) in aneurysmal and nonaneurysmal aortae. Frozen aortic sections were stained with (a) H&E, (b) EVG (elastin), and (c) SMC *α*-actin antibody (SMCs). Day 0: normal aorta with organized elastin and SMC layers and days 7, 14, and 28: aneurysmal aorta with medial elastin degradation, SMC loss, and leukocyte infiltration.

**Figure 3:**
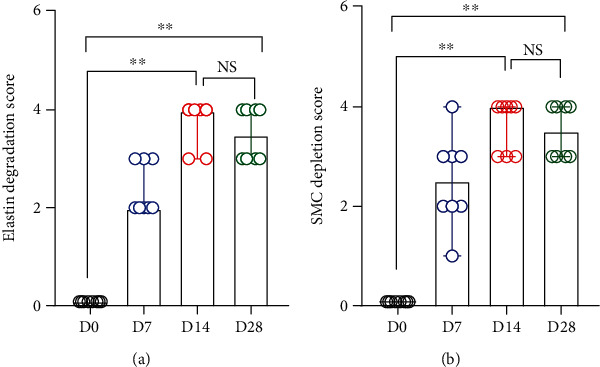
Quantification of medial elastin degradation and SMC depletion. Data are media and 95% CI of the scores for (a) medial elastin degradation and (b) SMC depletion. Nonparametric Kruskal-Wallis test, ^∗∗^*P* < 0.01 between two groups. *n* = 8 mice in each group. NS: not significant difference; D: day.

Seven days after PPE infusion, H&E stain showed disorganized aortic structure. Elastin degradation (score as 2 (2–3)) and SMC loss (score as 2.5 (1–4)) were also readily identified in EVG and SMC alpha-actin staining (Figure 2). Pronounced elastin degradation was found in aortae of mice 14 (score as 4 (3–4) (median and 95% CI)) and 28 (score as 3.5 (3–4)) days after PPE infusion, without significant differences noted between days 14 and 28 (Figure 3(a)). Similar results was found for SMC depletion, with the scores of 4 (3–4) and 3.5 (3–4) for days 14 and 28, respectively, again without differences between two time points (Figures 2 and 3(b)). These results suggested that, consistent with aortic enlargement, medial elastin degradation and SMC depletion achieved maximal 14 days after AAA induction.

### 3.3. Aortic Leukocyte Accumulation

**Figure 4:**
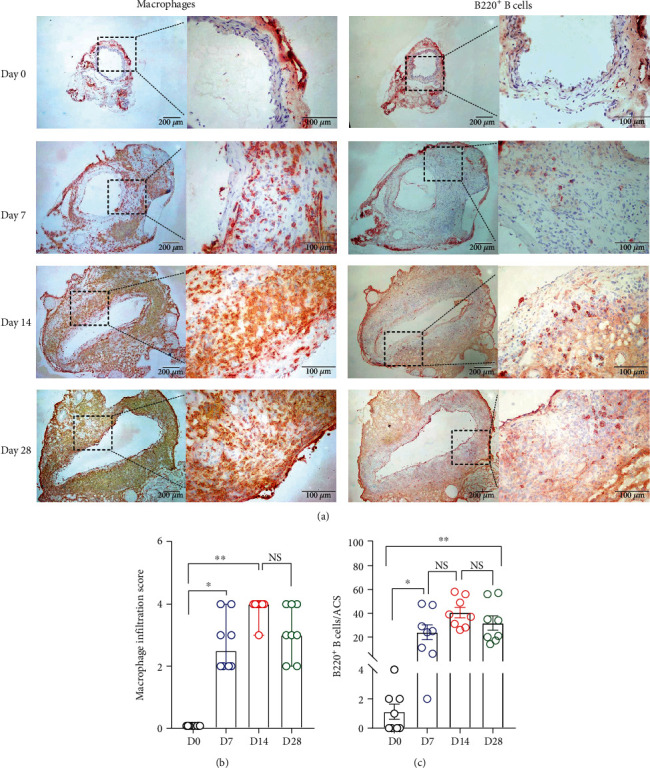
Accumulation of macrophages and B cells in nonaneurysmal and aneurysmal aortae. (a) Representative images for macrophages and B cells stained with mAbs against CD68 and B220 were used to check macrophages and B cell infiltration in aortic wall. Macrophage infiltration was graded as I (mild) to IV (severe) according to the abovementioned method. (b) Media and 95% CI of the scores for CD68-positive macrophage accumulation. (c) Mean and SE of B220^+^ B cells per aortic cross section (ACS). (b) Nonparametric Kruskal-Wallis test or one-way ANOVA followed by two-group comparison, ^∗^*P* < 0.05 and ^∗∗^*P* < 0.01 between two groups. *n* = 8 mice in each group. NS: not significant difference; D: day.

**Figure 5:**
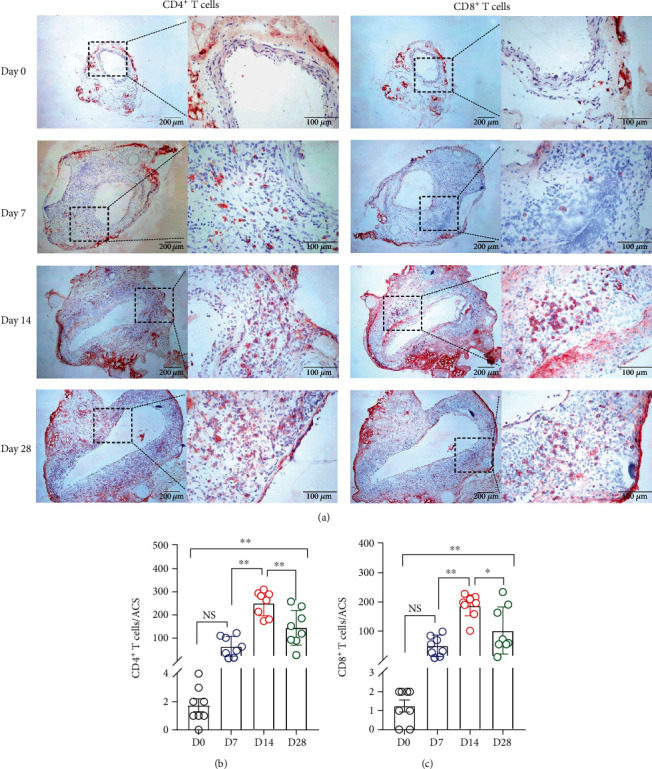
Accumulation of CD4^+^ T cells and CD8^+^ T cells in nonaneurysmal and aneurysmal aortae. (a) Representative images for CD4^+^ T cells and CD8^+^ T cells stained with mAbs against CD4 and CD8 antigens. (b, c) Mean and SE of (b) CD4^+^ T cells/aortic cross section (ACS) or (c) CD8^+^ T cells/ACS. One-way ANOVA followed by two-group comparison, ^∗^*P* < 0.05 and ^∗∗^*P* < 0.01. *n* = 8 mice in each group. NS: not significant difference; D: day.

In nonaneurysmal aorta, no or rare leukocytes were identified using subset-specific mAb immunostaining. In contrast, all subsets of leukocytes, including CD68^+^ macrophages, B220^+^ B cells, CD4^+^ T cells, and CD8^+^ T cells, were readily found at either time point examined following PPE infusion (Figures 4 and 5). Macrophages predominated over other leukocytes (Figure 4(b)). On day 7, macrophage (scored as 2.5 (2–4)) and B cell (24.0 ± 5.7 cells/ACS) accumulation, but not the count for CD4^+^ (63.1 ± 14.3 cells/ACS) or CD8^+^ (49.5 ± 12.2 cells/ACS), were significantly increased as compared to nonaneurysmal aorta (macrophage infiltration scores, 0; B cells, 1.4 ± 0.81 cells/ACS; CD4^+^ T cells, 1.8 ± 0.4 cells/ACS; and CD8^+^ T cells, 1.3 ± 0.3 cells/ACS, respectively) (Figures 4(b) and 4(c) and 5(b) and 5(c)). On day 14, the densities for all subsets of leukocytes (macrophage accumulation score, 3.9 ± 0.1; B cells, 40.5 ± 4.1 cells/ACS; CD4^+^ T cells, 250.9 ± 17.7 cells/ACS; and CD8^+^ T cells, 186.3 ± 13.5 cells/ACs, respectively) were significantly higher than those in nonaneurysmal aorta. There were also significant differences in densities for macrophages, CD4^+^ T cells, and CD8^+^ T cells, but not B cells, between days 7 and 14. On day 28, the densities for all leukocytes declined as compared to those on day 14 but were still significantly higher than those on day 0 (Figures 4 and 5). Leukocyte densities were 3.1 ± 0.3 (score), 31.8 ± 5.6 cells/ACS, 145.0 ± 26.0 cells/ACS, and 101.5 ± 27.1 cells/ACS for macrophages, B cells, CD4^+^ T cells, and CD8^+^ T cells, respectively, on day 28 following PPE infusion, with significant differences noted for CD4^+^ T cells and CD8^+^ T cells between days 14 and 28 (Figures 5(b) and 5(c)). Altogether, aortic accumulation of leukocytes peaks 14 days following PPE infusion and declines thereafter.

### 3.4. Mural Angiogenesis

**Figure 6:**
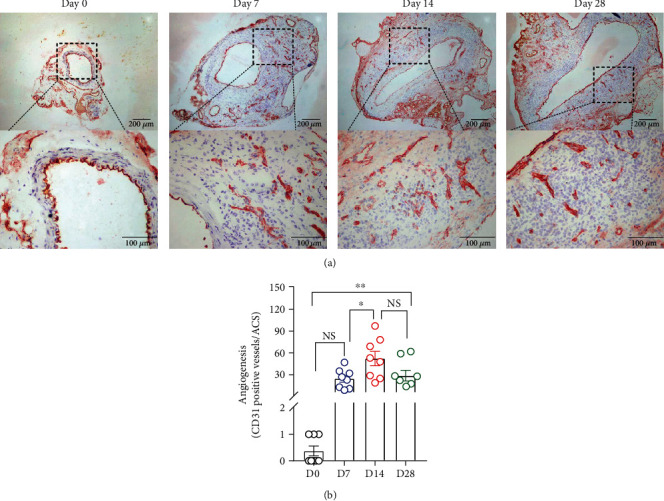
Mural angiogenesis in aneurysmal and nonaneurysmal aortae. (a) Representative images for neovessels identified by CD31 antibody staining. (b) Quantification of ceovessels (mean and SE of neovessels/aortic cross section (ACS)). One-way ANOVA followed by two-group comparison, ^∗^*P* < 0.05 and ^∗∗^*P* < 0.01 between two groups. *n* = 8 mice in each group. NS: not significant difference; D: day.

Augmented mural angiogenesis represents another pathological feature of AAA disease. In CD31 staining, no or rare neovessels were found in nonaneurysmal aorta (Figure 6(a)). Following PPE infusion, CD31 staining identified slight increase in neovessel density on day 7 (24.6 ± 4.4 vessels/ACS). Neovessel density reached maximal levels on day 14 (52.3 ± 9.2 vessels/ACS) and diminished on day 28 (28.9 ± 6.9 vessels/ACS), following AAA induction (Figure 6(b)). Neovessel densities on days 14 and 28 were significantly higher than that in nonaneurysmal aorta (Figure 6(b)). Thus, neovessel formation undergoes similar time-course as mural leukocyte accumulation.

### 3.5. Expression of MMP2 and MMP9

**Figure 7:**
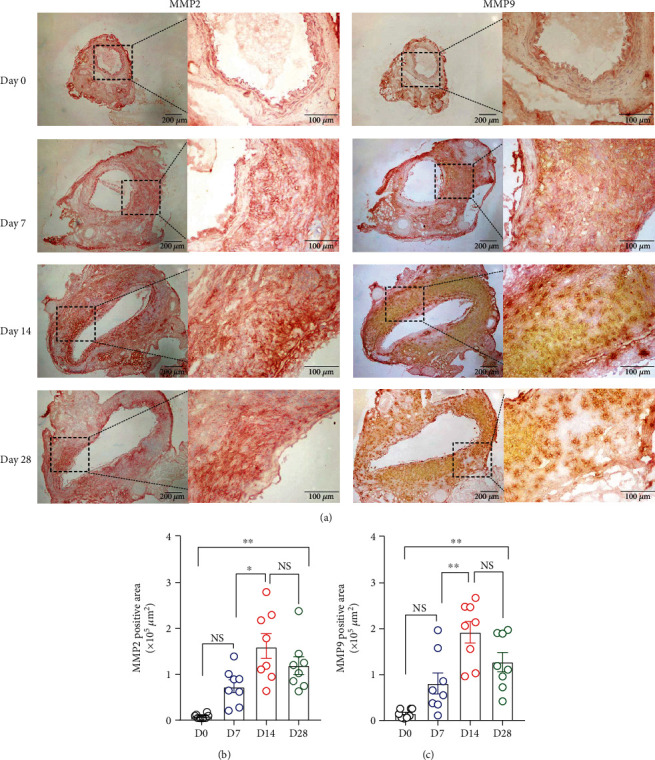
MMP2 and MMP9 expression in nonaneurysmal and aneurysmal aortae. (a) Antibodies against MMP2 and MMP9 were used to check MMP expression level. The CD4 or CD8 positive T cells were counted under microscope. Quantification of (b) MMP2 and (c) MMP9-positive staining areas (mean and SE). One-way ANOVA followed by two-group comparison was performed. *n* = 8 for each group; ^∗^*P* < 0.05 and ^∗∗^*P* < 0.01. NS: not significant; ACS: aortic cross section; D: day.

MMPs produced by macrophages, SMCs, and other cells are mediators leading to elastin and extracellular matrix destruction and thus aortic dilation. In immunostaining for MMP2 and MMP9, no remarkable staining for MMP2 or MMP9 was observed over background or negative control staining in nonaneurysmal aorta (Figure 7). Following AAA induction, either MMP2 or MMP9 expression was readily seen on day 7 (MMP2, 0.7 ± 0.1 × 10^5^ *μ*m^2^ and MMP9, 0.8 ± 0.2 × 10^5^ *μ*m^2^, respectively) and reached maximal on day 14 (MMP2, 1.6 ± 0.3 × 10^5^ *μ*m^2^ and MMP9, 1.9 ± 0.2 × 10^5^ *μ*m^2^, respectively) as compared to nonaneurysmal aorta (Figures 7(b) and 7(c)). Although both MMP2 and MMP9 expression levels declined on day 28, they were still significantly higher than that in nonaneurysmal aorta (Figures 7(b) and 7(c)). These data indicate that critical AAA proteinase production parallels key AAA histopathologies in elastase-induced mouse experimental AAAs.

## 4. Discussion

In clinical aneurysmal specimen analysis, AAAs are characterized by enlarged aortic diameter, decreased smooth muscle cells, broken or degraded elastin, digestion of extracellular matrix, and increased inflammation [21, 22]. With implementation of endovascular aneurysm repair, it is difficult to collect clinical AAA specimens for research purpose [23–25]. Animal models have become critically important for studying the pathogenesis and therapies of AAAs [12, 26–28]. While experimental AAAs can be induced in several rodents for biomedical and therapeutical studies, the mouse is the most popular used species [12, 26, 27, 29, 30]. A previous study, which focuses on spatiotemporal matrix remodeling, pointed out that aortic expansion was slower between 14 and 21 days than earlier, suggesting autostabilization of the AAA in rats [31]. However, no published data are available for time-course for aortic histopathologies in elastase-induced mouse experimental AAAs.

In this study using PPE-induced mouse AAAs, aortic diameter averagely reached 1.3 mm on day 14 after AAA surgery, which was 2.6 times of the baseline level. It has been shown that PPE infusion at high concentration increased aortic dilation with high mortality in mice [5, 32]. In this study, the aorta dilatation was faster from 1 to 14 days after the surgery, and it peaked 14 days after surgery. From day 14 to day 28 after the surgery, aortic diameter increased less, without statistical difference between two time points. Aortic dilation after PPE infusion was noted in two phases after the surgery: the first was during days 0-7 after the surgery, with moderate aortic expansion and acute inflammation; the second was days 7-14 after surgery, with remarkable elastin degradation, inflammatory cell infiltration, and MMP expression. Generally, aortic dilation in the PPE model is a combination of mechanical expansion and PPE stimulation during the first 3 days. Usually, after two weeks of PPE infusion, aortic dilation gradually reaches the plateau phase. Our results confirmed that 14 days after the surgery is the proper time point for histological evaluation of PPE-induced mouse AAA [8, 9].

Consistent with aortic diameter change, elastin degradation, SMC depletion, leucocyte infiltration, angiogenesis, and MMP expression also reached maximal during days 14-28. From days 0 to 7 after PPE infusion even more early, mild aortic expansion induced by both mechanical force and inflammation following PPE infusion was clearly visible, but aortic diameter has not yet met the criteria for aneurysm formation. On day 14 after the surgery, aortic diameter dilation reached the diagnostic criteria for AAAs, with characteristical aneurysmal histological changes. As aortic inflammation progresses gradually, aneurysmal tissue repair process may be initiated. Our results are consistent with previous studies in PPE-induced rat AAA models [31]. Our data suggest that day 14 after PPE infusion in mice is an important time point for histologically studying AAA pathogenesis and evaluating therapeutic efficacy.

Because our study focused on temporal changes in aneurysmal histopathologies, some limitations existed. First, aortic leukocyte cellularity was phenotyped based on surface marker expression. It was not assessed whether individual leukocytes undergo a functional switch. These include classical activated proinflammatory macrophages, alternatively activated anti-inflammatory macrophages, interferon-gamma- and interleukin-17-producing proinflammatory CD4^+^ and CD8^+^ T cells, and interleukin-10- and transforming growth factor-beta1-producing immunoregulatory T cells [33, 34]. Second, immunohistochemistry detected total MMP expression levels but was not able to differentiate active MMPs from pro-MMPs. Third, angiogenesis analysis was conducted for vessel density alone. Vessel phenotypes including vessel length, sprouting, and tortuosity were however not detailed. Additionally, it remained unclear whether residual SMCs in late phase of AAAs were dominated by contractible or secretory phenotype. Therefore, future investigations are necessitated to explore functional alternations in aortic immune cells, SMCs, and MMPs during AAA progression.

Similar to human AAA, the PPE model allows to create AAA in infrarenal aorta where almost clinical AAAs locate [5, 8, 12, 26, 27]. In this study, we provide the evidenced that day 14 after the surgery is sufficient for analyzing aneurysmal histopathologies in AAA biomedical and translational preclinical studies.

## Data Availability

Data can be available by contacting the corresponding author.
